# Controlled Polyelectrolyte Association of Chitosan and Carboxylated Nano-Fibrillated Cellulose by Desalting

**DOI:** 10.3390/polym13122023

**Published:** 2021-06-21

**Authors:** Sarah Amine, Alexandra Montembault, Matthieu Fumagalli, Anayancy Osorio-Madrazo, Laurent David

**Affiliations:** 1 Ingénierie des Matériaux Polymères IMP UMR 5223—CNRS, Université Claude Bernard Lyon 1, Université de Lyon, 69622 Villeurbanne, France; sarah13amine@gmail.com (S.A.); alexandra.montembault@recherche.univ-lyon1.fr (A.M.); matthieu.fumagalli@univ-lyon1.fr (M.F.); 2 Laboratory for Sensors, Institute of Microsystems Engineering—IMTEK, University of Freiburg, 79110 Freiburg, Germany; anayancy.osorio@imtek.uni-freiburg.de; 3 Freiburg Materials Research Center—FMF, University of Freiburg, 79104 Freiburg, Germany; 4 Freiburg Center for Interactive Materials and Bioinspired Technologies—FIT, University of Freiburg, 79110 Freiburg, Germany

**Keywords:** hydrogel, composites, chitosan, cellulose nanofibers, polyelectrolyte, desalting

## Abstract

We prepared chitosan (CHI) hydrogels reinforced with highly charged cellulose nanofibrils (CNF) by the desalting method. To this end, the screening of electrostatic interactions between CHI polycation and CNF polyanion was performed by adding NaCl at 0.4 mol/L to the chitosan acetate solution and to the cellulose nanofibrils suspension. The polyelectrolyte complexation between CHI polycation and CNF polyanion was then triggered by desalting the CHI/CNF aqueous mixture by multistep dialysis, in large excess of chitosan. Further gelation of non-complexed CHI was performed by alkaline neutralization of the polymer, yielding high reinforcement effects as probed by the viscoelastic properties of the final hydrogel. The results showed that polyelectrolyte association by desalting can be achieved with a polyanionic nanoparticle partner. Beyond obtaining hydrogel with improved mechanical performance, these composite hydrogels may serve as precursor for dried solid forms with high mechanical properties.

## 1. Introduction

Natural polymers such as polysaccharides are gaining interest and application niches over synthetic polymers for a variety of reasons. They are dispersible or soluble in aqueous solvents, they are non-toxic, issued from renewable sources, and can be degraded in different biological media.

However, in the particular field of biomaterials, polysaccharides are of major interest for their cytocompatibility and resorption as materials constituting implants. They exhibit a wide range of physico-chemical properties with specific biological behaviors and can be reproducibly processed in absence of organic solvent and toxic crosslinkers, the use of the latter complicating the marking procedures of medical devices.

Biomedical applications for chitosan (CHI) [1,2] and cellulose [3] have largely been investigated, and their association is also attractive [4,5,6].

In particular, chitosans with a low degree of acetylation (DA) and chitosan derivatives possess special interests for their healing properties, possibly due to (i) the ability of complexation with a variety of transition metal cations [7,8,9,10] (Cu^2+^, Co^2+^, Ni^2+^, Zn^2+^, Fe^3+^, etc.), contributing in vivo to the restoration of metallic homeostasis; (ii) the release of bioactive chito-oligosaccharides with chitosan degradation (with anti-inflammatory [11] and radical scavenger effects [12]); and (iii) binding with cells [13,14,15], proteins [16], extracellular matrices deposits, and mucus [17] through a variety of physical interaction types: electrostatic interactions when CHI is partly protonated or hydrogel bonds or hydrophobic interactions when CHI is in its neutralized free amine form at pH above 6.2. Chitosan is currently used as hydrogel (*Chitogel* for sinus surgery, Medtronics) and solution (*Cargel Bioscaffold* for cartilage repair, Smith and Nephew) but other possible physical forms are of interest, i.e., reinforced hydrogels, fibers, and films, for applications where good mechanical properties may be required, such as suture threads or knitted fabrics for parietal implants. Thus, there is still a need to develop CHI-based materials with improved mechanical properties. Different strategies have been explored to optimize the mechanical properties of CHI biomaterials. A first approach is based on the control and orientation of the crystallinity (crystallinity ratio, type of allomorph, crystalline orientation) through the fine tuning of polymer processing and formulation [18,19,20]. A second possibility is to use nanofillers, in particular nanofibrils, to reinforce a chitosan matrix. In the context of biomaterials, successful approaches were proposed to introduce resorbable fibrillar fillers such as collagen fibrils [21], chitosan nanofibrils [22,23,24], or cellulose nanofibrils [25] in chitosan solids or hydrogels.

Cellulose nanofibrils also exhibit a wide range of morphologies and can be found and extracted from biomass in the form of elongated crystals (cellulose nanocrystals or cellulose whiskers) [26,27] or semicrystalline interconnected fibrils known as micro-/nano-fibrillated cellulose (CNF) [28,29,30]. Such nano-objects can be redispersed in aqueous media to form suspensions or hydrogels with good cytocompatibility for applications in tissue engineering [31,32,33]. As a colloid or nanofiller, the physico-chemical and mechanical properties of microfibrillated cellulose are strongly impacted by the aspect ratio of the fibrils, their degree of interconnection, and the negative charge carried on the surface of the nanofibrils. Sulfuric acid treatments during the extraction process are known to introduce sulfate groups [34], but oxidation treatments (e.g., TEMPO-mediated oxidation [35]) can bring carboxylic moieties at the surface. Introducing high charge density onto cellulose nano-fibrils favors their dispersion in aqueous suspensions, but could also promote interactions with polycations, the charged nanofibril acting as a polyanion to form polyelectrolyte complexes [32].

The association of chitosan with cellulose was addressed in the case of cellulose-rich materials [36] where chitosan chains are adsorbed onto the cellulose network by electrostatic interactions (when chitosan is in the hydrophilic protonated state) and H-bonds and hydrophobic interactions when chitosan is further neutralized by an alkaline treatment. Such interaction tuning results in a strong increase of the apparent modulus of films after casting, even in the presence of water. In the case of chitosan-rich systems, nanofibrillated cellulose-reinforced chitosan hydrogels were successfully obtained using a weakly charged nanocellulose and ultrasonic treatment of a pre-mixture to ensure a satisfactory dispersion of the nanofibrils within the chitosan solution, before gelation of chitosan with a base [31,32]. The possibility to tune the mechanical properties of hydrogels, at subcellular scale, with the possibility to prepare soft materials in a wide elastic modulus range appears to be key to control the fate of cells at their contact [37], since cell phenotype may be impacted by the mechanical cues of their micro-environment. The stretching and drying of such nanocomposite hydrogels yields solid materials with inherited improved mechanical properties, in particular in the case of yarns [38]. Hence, like in other polysaccharide polyelectrolyte systems, the difficulty of ensuring a molecular dispersion of both polycation (chitosan) and polyanion(s) for an optimal polyelectrolyte interaction remains a challenge. In this work, we performed highly anionic CNF and CHI polyelectrolyte associations by desalting CHI/CNF suspensions. With the electrostatic interactions being screened by the presence of salts, we thus performed a controlled polyelectrolyte complexation, initially designed for soluble polyelectrolytes [39,40,41].

## 2. Materials and Methods

### 2.1. Chitosan Sourcing and Characterization

Chitosan from squid pen chitin was supplied by Mahtani Chitosan (Veraval, India; batch type 144). ^1^H NMR analysis was used to determine the degree of acetylation (DA) close to 4.0 ± 0.5% following the methodology of Hirai et al. [42]. A weight-average molar mass Mw of 580 ± 50 kg/mol and a dispersity Đ of 1.5 ± 0.3 were determined as previously described [43], using size exclusion chromatography (SEC) coupled online with a differential refractometer (Optilab T-rEX, Wyatt; λ = 658 nm) and with a multi-angle laser light scattering (SEC-MALLS) detector (Dawn-HELOES II, Wyatt; λ = 664 nm) at the Chromatography Center of Institute of Chemistry of Lyon (ICL).

*Preparation of chitosan acetate solutions:* Chitosan powder was dispersed in deionized water. Then, a stoichiometric amount of acetic acid vs. amine groups was added to protonate chitosan, accounting for a DA value of 4% and a water content close to 8% *w*/*w*, determined by thermogravimetry analysis (mass loss between 30 °C and 200 °C). This acetic acid amount allowed complete solubilization of chitosan under mechanical stirring in a closed reactor for 12 h. Chitosan solutions were obtained at different concentrations, ranging from 1.7% *w*/*w* to 5% *w*/*w*.

### 2.2. Cellulose Nanofibers

Carboxylated nanofibrillated cellulose suspensions were provided by the Centre Technique du Papier (CTP, Grenoble, France). These suspensions were obtained from a bisulfite birch pulp which was pretreated with TEMPO to introduce surface carboxylic groups, and nanofibrils were subsequently mechanically individualized through two passes in a homogenizer at 1500 bar. The resulting suspension had a gel like texture and a solid content of 1.2 % *w*/*w*. The suspension diluted in deionized water at a concentration of 0.44% *w*/*w* was further purified by dialysis against deionized water using a membrane with cut-off of 6–8 kD, until the conductivity of the dialysis bath was constant close to 0.9 mS/cm.

*Carboxylate content of CNF:* The carboxylate content of the nanofibrillated cellulose was determined by conductimetric titration with NaOH solution in the presence of an excess of hydrochloric acid (pH ~2.5). The low conductivity plateau Δ*V* was measured and allowed to calculate the ionic exchange capacity [18]. The carboxylate content can also be expressed as the molar ratio of oxidized repeat units of cellulose, i.e., degree of oxidation (*DO*) and calculated from:

(1) $$DO=\frac{m_{AGU}\times \Delta V\times C_{NaOH}}{m-(m_{ox\times AGU}-m_{AGU})\times \Delta V\times C_{NaOH}}$$

where *m_AGU_* = 162 g/mol is the molar mass of the anhydroglucose repeat unit of cellulose, *m_Ox_._AGU_* = 198 g/mol is the molar mass of oxidized repeat unit, *C_NaOH_* is the molar concentration of NaOH used for the titration, Δ*V* = *V*_2_ − *V*_1_ is the volume extent of the low conductivity plateau, and *m* (g) is the mass of the dry CNF sample. The value of the *DO* was close to 0.22 mol/mol and the corresponding equivalent ionized carboxylate content (ion exchange capacity) of 1.3 meq/g. For hardwood pulp, it was reported that the reaction reached a threshold at 1.6 meq/g corresponding to the oxidation of all the primary hydroxyls lying on the surface of the nanofibrils. Herein, about 75% of the primary surface hydroxyls were oxidized to carboxylate.

*Attenuated total reflection Fourier transform infrared spectroscopy (ATR-FTIR):* ATR-FTIR analyses were performed on dry CNF films. FTIR spectra were recorded with a Thermoscientific iS–10 operating in ATR mode with a germanium crystal (64 scans, wavenumber range 670 to 4000 cm^−1^, normal contact, resolution of 4 cm^−1^).

Transmission electron microscopy observation of CNFs: Drops of about 0.001 wt% of aqueous suspension of CNCs were deposited onto glow-discharged carbon-coated 3 mm grids for transmission electron microscopy (TEM). After 2 min, the liquid in excess was wicked off with a filter paper and a drop of Uranyless staining solution was deposited on the specimen. After 2 min, the stain in excess was wicked off and the remaining thin liquid film allowed to dry. The specimens were observed with a Philips CM120 CRYO TEM operated at 120 kV.

### 2.3. Rheological Study

The viscoelastic properties of mixtures of CNF suspensions and chitosan solutions as well as hydrogels produced after neutralization in NaOH solution 1 M were studied by dynamic-mechanical rheological measurements. These measurements were carried out using an ARES rheometer (TA Instruments) operating with a plate-plate geometry (diameter of 25 mm) at room temperature. The strain amplitude was monitored to ensure the measurements, which were carried out within the linear viscoelastic region, resulting in a storage modulus (*G*′) and loss modulus (*G*″) independent of the strain amplitude. Thus, we carried out angular frequency sweep measurements at an applied strain of 1%. We repeated these analyses at least three times for each type of hydrogel.

## 3. Results and Discussion

### 3.1. Stability of Chitosan (CHI) Solution and CNF Suspension in the Presence of NaCl Salt

**Figure 1:**
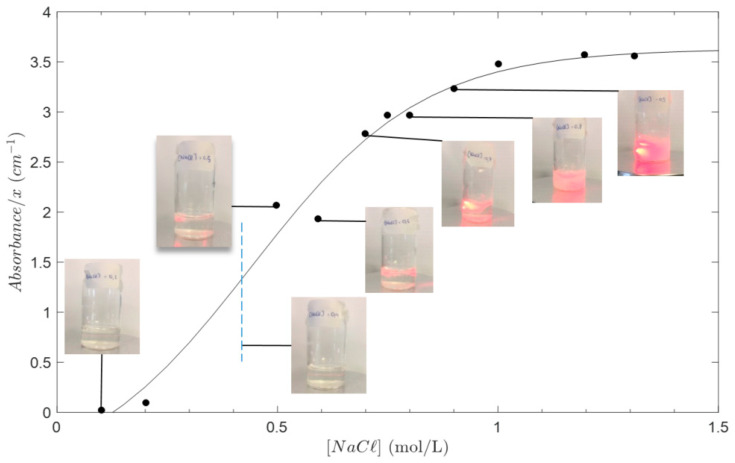
Solubility study of chitosan acetate solutions with added NaCl. The concentration of chitosan was 3% *w*/*w*. Solubility is quantified by the evolution of absorbance at 400 nm (adapted from [40]), and precipitation is displayed by images of the samples submitted to a laser beam (λ = 650 nm). Solubility of chitosan acetate in saline aqueous solution was limited to the NaCl concentration range from 0 to 0.4 M.

Figure 1 shows the evolution of suspensions of chitosan with increasing NaCl content, for a solution at a chitosan concentration of 3% (*w*/*w*). Screening effects at high salt concentration induces a precipitation of chitosan [40]. Thus, the accessible NaCl concentration was restricted to be below 0.4 M. Above this limit, the solution became highly turbid and scattered light at 650 nm and 400 nm.

### 3.2. Cellulose Nanofibers (CNF) Microstructure and Stability of Suspensions in the Presence of NaCl Salt

**Figure 2:**
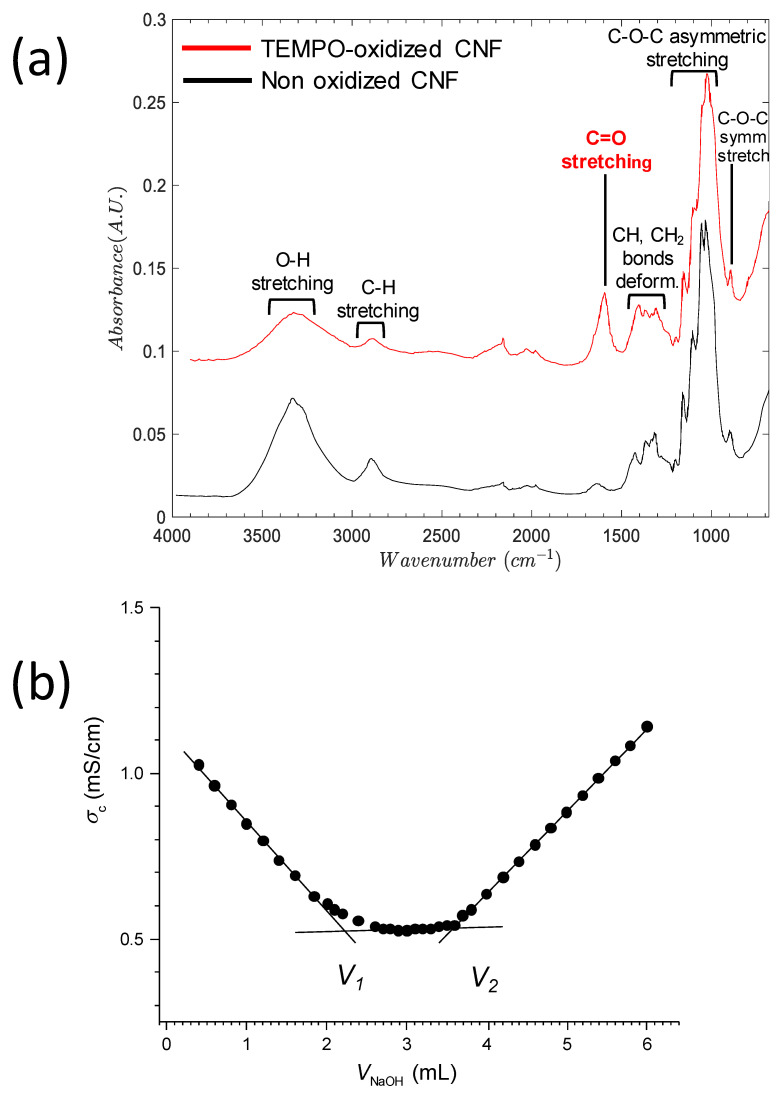
(**a**) ATR-FTIR spectra of the starting non-oxidized and the TEMPO-oxidized cellulose nanofibers. (**b**) Determination of the degree of oxidation (DO) by electric conductivity titration method. The corrected conductivity *σ_c_* was related to the measured conductivity *σ_m_* according to $\sigma_{c}=\sigma_{m}(V_{i}+V_{NaOH})/V$ where *V*_i_ (= 83 mL) was the initial volume of the acidic CNF suspension (*m* = 0.102 g, pH = 2.5) and *V*_NaOH_ is the added NaOH volume (*C*_NaOH_ = 1 M). The value of DO was deduced from parameter Δ*V* = *V*_2_ − *V*_1_ (V_2_ = 3.59 mL, V_1_ = 2.28 mL) according to Equation (1).

The chemical structure of the TEMPO oxidized CNF and the starting non-oxidized cellulose nanofibers were investigated by ATR-FTIR (Figure 2a). The spectra displayed a broad absorption band around 3350–3150 cm^−1^, corresponding to the O–H stretching, which indicates the formation of intermolecular H-bonds in both nanofiber samples. The peaks at around 2850–2930 cm^−1^ are attributed to the asymmetric and symmetric stretching vibrations of the C–H group, respectively. In the non-oxidized CNF, absorption around 1635 cm^−1^ was observed, attributed to -OH bending [44]. The ATR-FTIR spectrum of the TEMPO-mediated oxidized CNF showed the specific band of the C=O stretching signal, around 1600 cm^−1^, evidencing the presence of carboxylate ions in the TEMPO-oxidized CNFs [45]. Then, the deformation vibrations of the CH, CH_2_ groups were visible around 1300–1400 cm^−1^ in both spectra, but with reduced intensity of the asymmetric deformation of methylene normally appearing higher than 1400 cm^−1^ in the spectrum of TEMPO-oxidized CNF as expected after carboxylation of the CH_2_OH groups of cellulose. Then, the asymmetric stretching of the C–O from primary, secondary alcohol, and (hemi)acetal groups presented their characteristic bands at 1000–1300 cm^−1^, and the C-O-C symmetric stretching at around 800–900 cm^−1^. Finally, the fingerprint pattern of polysaccharides was clearly observed at the range between 1300 and 800 cm^−1^.

**Figure 3:**
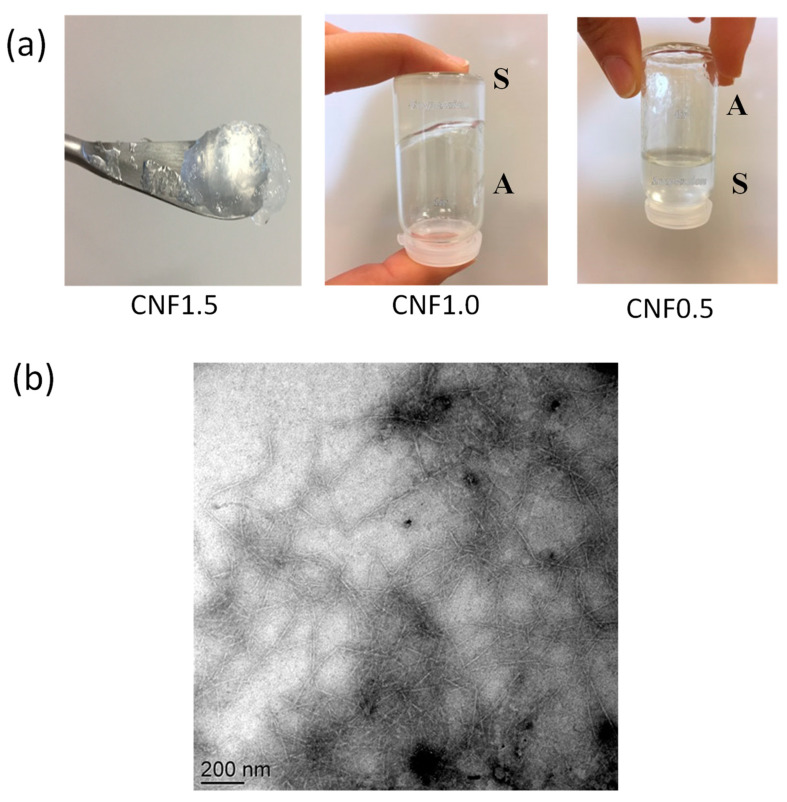
(**a**) Photos showing the appearance and flow of viscous suspensions containing TEMPO-oxidized cellulose nanofibers (CNF) at different concentrations of 1.5, 1.0, and 0.5 % *w*/*w*. S: Solution/suspension, A: air. (**b**) TEM micrograph of TEMPO-oxidized cellulose nanofibers.

Figure 3a displays the gel aspect of CNF suspensions. The tube inversion test showed a gel-like behavior for concentrations higher than ~1% *w*/*w*, as a result of the formation of a network of interacting cellulose fibrils.

The TEM micrograph of the TEMPO-oxidized CNF (Figure 3b) shows well individualized fibrils of 200–300 nm length and 6–8 nm width, in good agreement with previous reports from the literature [35].

**Figure 4:**
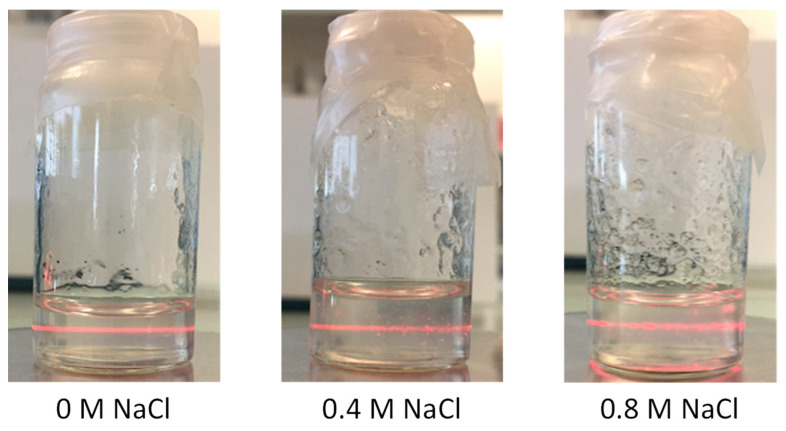
Laser light scattering observation (λ = 650 nm) for a qualitative evaluation of turbidity of 0.45% *w*/*w* CNF suspensions with different NaCl concentrations. The suspensions at pH = 5.1 were not significantly affected by the presence of NaCl salt in the range 0 to 0.8 M.

CNF suspension stability (pH = 5.1) was qualitatively studied as shown in Figure 4. Laser scattering appeared similar in the NaCl concentration range from 0 to 0.8 M. As a result, in order to obtain the highest screening effect without precipitation, a NaCl concentration of 0.4M could be determined. In these conditions, the stability of salted CNF suspensions and CHI solutions were obtained over 1 week.

Lastly, we investigated the impact of the pH of CNF suspensions and CHI solutions on their turbidity. Gelation of CNF suspensions occurred at pH ~3 and below, in good agreement with the pKa range of glucuronic acids (typically between 2.7 to 3.3) [46,47]. The pH of CHI solutions ranged from 5.1 to 4 with concentrations ranging from 1.5 to 5% *w*/*w*. The stability of CHI was preserved up to a pH close to 5.8, a value slightly below the apparent pKa ~6.2 of CHI at low DA [48]. Thus, the pH window for the association of chitosan and CNF without precipitation was located between their pKa values, typically between 4 and 5.5. As a result, in order to mix the CHI solution with the CNF suspension, the pH and NaCl concentration of both media should stay in a well-defined processing window.

### 3.3. Formulation and Desalting of Polyelectrolyte Mixtures of CHI Solutions and CNF Suspensions

We easily prepared homogeneous CHI/CNF mixtures by imposing a pH of 4 both on the CHI solution and on the CNF suspension by the addition of a diluted acetic acid solution. For a proof of concept, the values of the CHI and CNF concentration tested were chosen below the gelation threshold in both cases. This ensured excellent mixing conditions with magnetic stirring, but higher concentrations are assessable for both components with mechanical blending. The resulting systems are described in Table 1. As expected, when comparable systems were prepared without NaCl addition, precipitation resulted in a macroscopically heterogeneous system with no satisfactory dispersions of CNF within the chitosan solution.

Desalting of final mixtures with a 6–8 kD cut-off membrane could not be performed directly against deionized water, since it induced a large dilution of the samples. To minimize the effect of reverse osmosis, we used a 5-staged dialysis (engaging about 10 mL of salted polyelectrolyte mixture) against NaCl aqueous solutions (500 mL) with concentrations of 0.32 M, 0.24 M, 0.16 M, 0.08 M, and 0 M, successively, each step of dialysis lasted 4 h, 16 h, 4 h, 4 h, and 16 h respectively. This strongly limited the dilution effects to a constant net water gain of 15% (*w*/*w*). The systems remained transparent after dialysis. Then, complete gelation was induced by submerging the dialysates in NaOH 1 M for 12 h, and further water washing for 24 h. Syneresis was negligible (as in the pure chitosan hydrogels of same low concentration, low DA, and high mean molar masses) and thus, the washed gels were obtained using *C*_CHI_ = 1.01% and *C*_CNF_ ranging from 0 to 0.15%.

### 3.4. Viscoelastic Properties of Resulting Hydrogels

**Figure 5:**
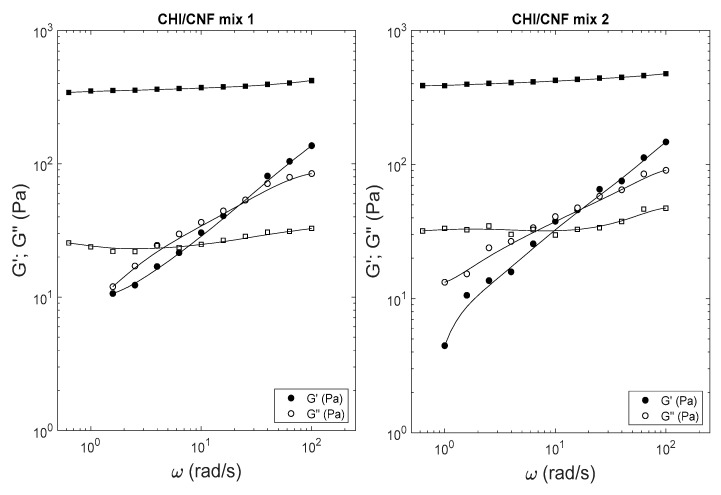
Viscoelastic properties of CHI/CNF associations obtained from mixtures 1 and 2 (Table 1) as *G*′; *G*″ sweeps of angular frequency *ω*: Samples were characterized after mixing of salted solutions and suspensions (circles) (G′: ●; G″: ○) and after multistep dialysis and gelation in NaOH 1 M (squares) (G′: ■; G″:□).

Figure 5 displays the viscoelastic behavior of the mixtures 1 and 2 before dialysis (circles) and after neutralization in the alkaline bath (squares). Similarly, all formulated systems exhibited a solution-like behavior after mixing in presence of NaCl, but exhibited a gel behavior after desalting and neutralization, with G′ >> G″ in the entire investigated angular frequency range.

**Figure 6:**
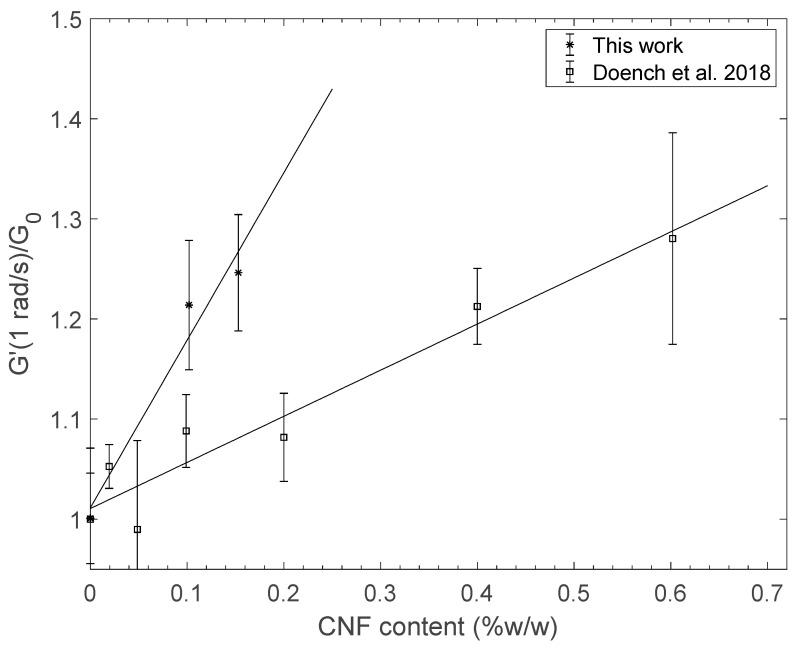
(*): Evolution of the elastic part of the shear modulus of hydrogels obtained from the controlled polyelectrolyte assembly of CHI and highly charged CNF by desalting, in normalized scales. The value of *G*_0_ for non-reinforced systems obtained in this work was close to 0.31 kPa. These results are compared with literature data (□) for CHI/CNF hydrogels obtained with weakly charged CNF [32].

A comparison of the elastic shear moduli of hydrogel samples prepared from the mixtures detailed in Table 1 is given in Figure 6. The reinforcement effect is clear as the elastic modulus increased with CNF content. Such behavior was previously analyzed [32] through the Einstein’s generalized relation (Equation (2)):

(2) $$G\prime =G_{0}(1+k_{E}\varphi_{v}{}_{,CNF})\approx G_{0}(1+k_{E}\frac{\varphi_{m}{}_{,CNF}}{\rho_{CNF}/\rho_{H20}})$$

where *G*_0_ is the value of the modulus of the non-reinforced reference system, *φ_v,CNF_* is the volume fraction of CNF filler, and *φ_m,CNF_* = *C*_CNF_ is the mass fraction of CNF filler, *ρ*_CNF_ ~ 1.43 g/cm^3^ is close to the density of nanofibrilated cellulose [49], and *k*_E_ is the Einstein coefficient (~2.5 for spherical objects) quantitatively accounting for the reinforcement effect. The value found for *k*_E_ was close to 120, corresponding to an extremely strong reinforcement effect. Since the CNF were not oriented by the hydrogel processing technique, the reinforcement effect should be related to crosslinking of the chitosan matrix by nanofibrils, as a result of CHI and CNF physical interactions. Chitosan in hydrogel samples is neutralized, hence its polyelectrolyte character is not directly responsible for these interactions. Nevertheless, electrostatic interactions may be understood as the first driving force for chitosan adsorption onto CNF nanofibers during the polyelectrolyte complexation. After neutralization of the complex, H-bonding and hydrophobic interactions must take over to ensure physical crosslinking, as is the case for the complexation of chitosans with other macromolecular polysaccharide polyanions [50]. In agreement with previous studies [31,32,36], the morphology of reinforced CNF/CHI hydrogels obtained in this work can be schematized as (i) a first fibrillar nanocellulose network resulting from H-bonds between nanofibers and chitosan chains bridging nearby nanofibers, and (ii) a second chitosan hydrogel network where isolated fibrils portions play the role of physical crosslinks of high functionality for chitosan chains. The formation of CHI/CNF interaction in both networks is driven by electrostatic interactions, that are in turn controlled by the charge density of the initial polyelectrolytes, i.e., by the degree of acetylation (DA) of chitosan and the degree of oxidation (DO) of nanocellulose. After the neutralization of chitosan, the remaining interactions of chitosan adsorbed onto the CNF nano-rods are due to H-bonds and hydrophobic interactions.

The reinforcement effects of CNF/CHI hydrogels may also be compared to pure CHI hydrogels at same total polymer concentration. The elastic shear modulus of CHI hydrogels may vary with mass concentration according to:

(3) $$G\prime =G_{00}{\left(C_{CHI}\right)}^{\beta }$$

where *G*_00_ should be close to the measured value at *C*_CHI_ = 1% *w*/*w* (i.e., *G*_0_ ~ 0.31 kPa) and *β* is an exponent previously found close to 4.4 by AFM force spectroscopy [51] for a chitosan of same mean molar mass and DA. Our rheological analyses combined with previous viscoelastic measurements [52] (see Sample H3b: *C*_CHI_ = 1.6% *w*/*w*, E = 6 kPa; sample H4: *C*_CHI_ = 2.6% *w*/*w*, *E* = 31 kPa, and assuming *E* = *G*/3, i.e., the Poisson’s coefficient ν = 0.5) yield similar values (G_00_ = 0.29 kPa, β = 4.15, *R*^2^ = 0.9998) after a linear regression in logarithmic scales. As a result, for a pure CHI hydrogel 1.2% *w*/*w*, *G*’/*G*_00_ > 2 and the resulting shear modulus ratio *G*’/*G*_0_ should largely exceed the values displayed in Figure 6. This can be explained, at a macromolecular level, by the involvement of all chitosan chains contributing to the CHI-CHI network via H-bonds, hydrophobic interactions, and the formation of nanocrystallites [53]. On the other hand, CNF physical crosslinking with CHI only occurs at the surface of the nanofibrils. Indeed, cellulose chains entrapped within the cellulose nanofiber cores should not contribute to crosslinking with the matrix: considering a given mass of polymer, the reinforcement effect of CNF in CHI hydrogels stays below the effect of CHI concentration. However, for materials obtained in the dry state, chitosan concentration of the parent hydrogel does not significantly impact the final hydration, density, interactions, and microstructure. As a result, composite reinforcement effects of CNF are more apparent in the solid (dry) state. Thus, the interest of CHI/CNF associations is particularly attractive in the case of films or fibers prepared from CHI/CNF associations [38].

## 4. Conclusions

In this work, we designed a controlled polyelectrolyte association between highly charged nanofibrillated cellulose (CNF) and chitosan (CHI) of low DA in excess. It required evaluating the stability of CNF suspensions and CHI solutions in the presence of a screening salt (NaCl) and the use of a multiple step dialysis against aqueous solutions with decreasing salt concentrations.

In comparison with previous literature results, we obtained hydrogels with higher mechanical properties using a highly charged CNF vs. weakly charged cellulose nanofibrils [32]. This effect should reflect the high physical crosslink capacity of highly charged CNF interacting with chitosan chains adsorbed onto their surface, thanks to the initial polyelectrolyte complexation of CHI and CNF.

Future studies should focus on the preparation strategies of systems at higher concentrations (for CHI and CNF), including the processing of dry fibers or films, where the composite reinforcement effects are expected to yield higher mechanical properties. In particular, yarns prepared by stretching and drying reinforced hydrogels should yield bioinspired solid materials with enhanced mechanical properties, with practical interest in the field of textile knitting.

## Figures and Tables

**Table 1:** Parameters of formulation used for the preparation of CHI/CNF mixtures from precursors CHI acetate solutions and CNF suspensions, and of the resulting formulations of two CHI/CNF mixtures, before dialysis and gelation. *C*_CHI_: mass concentration of chitosan (CHI); *C*_CNF_: mass concentration of nanofibrilated cellulose.

Sample Type	CHI Reference Solution	CHI-CNF Mix 1	CHI-CNF Mix 2
Precursor CNF suspension (30%)	Acetic Acid/NaCl solution without CNF, pH = 4 *C*_NaCl_ = 0.4 M	CNF/NaCl *C*_CNF_ = 0.4% pH = 4 *C*_NaCl_ = 0.4 M	CNF/NaCl *C*_CNF_ = 0.6% pH = 4 *C*_NaCl_ = 0.4 M
Final mixes	*C*_CHI_ = 1.19% *w*/*w* *C*_CNF_ = 0% *w*/*w* *C*_NaCl_ = 0.4 M pH = 4	*C*_CHI_ = 1.19% *w*/*w* *C*_CNF_ = 0.12% *w*/*w* *C*_NaCl_ = 0.4 M pH = 4 [NH3^+^]/[COO^−^] = 47	*C*_CHI_ = 1.19% *w*/*w* *C*_CNF_ = 0.18% *w*/*w* *C*_NaCl_ = 0.4 M pH = 4 [NH3^+^]/[COO^−^] = 31

## Data Availability

Not applicable.
